# Supplementary material for: A novel family of beta mixture models for the differential analysis of DNA methylation data: An application to prostate cancer
Source: PLoS One. 2024 Dec 11;19(12):e0314014. doi: 10.1371/journal.pone.0314014 (PMC11633993; doi:10.1371/journal.pone.0314014)
Supplement: S1 File — Supporting Information (Appendices S1 to S18) includes BMM derivations, parameter estimates and density estimate plots when BMM is applied to simulated and prostate cancer datasets. (PDF) [file pone.0314014.s001.pdf]

Supporting Information 1 for 'A novel family of beta mixture models for the differential analysis of DNA methylation data: an application to prostate cancer' data by Majumdar et al.

Appendix S1

K·· Model The complete data log-likelihood for this model is,

$$\ell_C(\boldsymbol{\tau}, \boldsymbol{\theta}, \mathbf{Z}|\mathbf{X}) = \sum_{c=1}^{C}\sum_{k=1}^{K} z_{ck}\{\log \tau_k + \sum_{n=1}^{N}\sum_{r=1}^{1} \log[\text{Beta}(x_{cnr}; \alpha_{k\cdot\cdot}, \delta_{k\cdot\cdot})]\}.$$

In the Expectation-step of the EM algorithm the $\hat{z}_{ck}$ is calculated given the current parameter estimates. In the Maximisation-step the expected complete data log-likelihood function to be optimized is,

$$\begin{aligned}\ell_C(\boldsymbol{\tau}, \boldsymbol{\theta}|\mathbf{X}, \hat{\mathbf{Z}}) = &\sum_{c=1}^{C}\sum_{k=1}^{K} \hat{z}_{ck}\{\log \tau_k + \\ &\sum_{n=1}^{N}\sum_{r=1}^{1}[(\alpha_{k\cdot\cdot} - 1)\log x_{cnr} + (\delta_{k\cdot\cdot} - 1)\log(1 - x_{cnr}) - \log \mathrm{B}(\alpha_{k\cdot\cdot}, \delta_{k\cdot\cdot})]\}.\end{aligned} \tag{1}$$

Differentiating (1) w.r.t $\alpha_{k\cdot\cdot}$ yields,

$$\frac{\partial \ell_C}{\partial \alpha_{k\cdot\cdot}} = \sum_{c=1}^{C} \hat{z}_{ck}\{\log x_{cnr} - [\psi(\alpha_{k\cdot\cdot}) - \psi(\alpha_{k\cdot\cdot} + \delta_{k\cdot\cdot})]\} \tag{2}$$

where ψ is the digamma function.

Similarly, the derivative of $\ell_C(\boldsymbol{\tau}, \boldsymbol{\theta}|\mathbf{X}, \hat{\mathbf{Z}})$ w.r.t $\delta_{k\cdot\cdot}$ is,

$$\frac{\partial \ell_C}{\partial \delta_{k\cdot\cdot}} = \sum_{c=1}^{C} \hat{z}_{ck}\{\log(1 - x_{cnr}) - [\psi(\delta_{k\cdot\cdot}) - \psi(\alpha_{k\cdot\cdot} + \delta_{k\cdot\cdot})]\}. \tag{3}$$

The lower bound value of the digamma function ($\psi(y) > \log(y - 1/2)$) is used in (2) and (3) to get closed-form solutions at the Maximisation-step of the EM algorithm,

$$\frac{\partial \ell_C}{\partial \alpha_{k\cdot\cdot}} \approx \sum_{c=1}^{C} \hat{z}_{ck} \sum_{n=1}^{N}\sum_{r=1}^{1}\left[\log x_{cnr} - \log \frac{\alpha_{k\cdot\cdot} - 1/2}{\alpha_{k\cdot\cdot} + \delta_{k\cdot\cdot} - 1/2}\right] \tag{4}$$

and

$$\frac{\partial \ell_C}{\partial \delta_{k\cdot\cdot}} \approx \sum_{c=1}^{C} \hat{z}_{ck} \sum_{n=1}^{N}\sum_{r=1}^{1}\left[\log(1 - x_{cnr}) - \log \frac{\delta_{k\cdot\cdot} - 1/2}{\alpha_{k\cdot\cdot} + \delta_{k\cdot\cdot} - 1/2}\right]. \tag{5}$$

Equating (4) and (5) to zero, we get the approximate estimates of $\alpha_{k\cdot\cdot}$ and $\delta_{k\cdot\cdot}$ as,

$$\alpha_{k\cdot\cdot} = 0.5 + \frac{0.5\exp(-y_2)}{\{[\exp(-y_2) - 1][\exp(-y_1) - 1]\} - 1}$$

and

$$\delta_{k\cdot\cdot} = \frac{0.5\exp(-y_2)[\exp(-y_1)-1]}{\{[\exp(-y_2)-1][\exp(-y_1)-1]\}-1},$$

where $y_1 = (\sum_{c=1}^{C} z_{ck} \log x_{cnr})/(N \sum_{c=1}^{C} z_{ck})$ and $y_2 = (\sum_{c=1}^{C} z_{ck} \log(1-x_{cnr}))/(N \sum_{c=1}^{C} z_{ck})$.

Appendix S2

KN· Model The complete data log-likelihood for this model is,

$$\ell_C(\boldsymbol{\tau}, \boldsymbol{\theta}, \mathbf{Z}|\mathbf{X}) = \sum_{c=1}^{C}\sum_{k=1}^{K} z_{ck}\{\log \tau_k + \sum_{n=1}^{N}\sum_{r=1}^{1} \log[\text{Beta}(x_{cnr}; \alpha_{kn\cdot}, \delta_{kn\cdot})]\}.$$

In the Expectation-step of the EM algorithm the $\hat{z}_{ck}$ is calculated given the current parameter estimates. In the Maximisation-step the expected complete data log-likelihood function to be optimized is,

$$\ell_C(\boldsymbol{\tau}, \boldsymbol{\theta}|\mathbf{X}, \hat{\mathbf{Z}}) = \sum_{c=1}^{C}\sum_{k=1}^{K} \hat{z}_{ck}\{\log \tau_k + \sum_{n=1}^{N}\sum_{r=1}^{1}[(\alpha_{kn\cdot} - 1)\log x_{cnr} + (\delta_{kn\cdot} - 1)\log(1 - x_{cnr}) - \log \mathrm{B}(\alpha_{kn\cdot}, \delta_{kn\cdot})]\}. \tag{6}$$

Differentiating (6) w.r.t $\alpha_{kn\cdot}$ yields,

$$\frac{\partial \ell_C}{\partial \alpha_{kn\cdot}} = \sum_{c=1}^{C} \hat{z}_{ck}\{\log x_{cnr} - [\psi(\alpha_{kn\cdot}) - \psi(\alpha_{kn\cdot} + \delta_{kn\cdot})]\} \tag{7}$$

where ψ is the digamma function.

Similarly, the derivative of $\ell_C(\boldsymbol{\tau}, \boldsymbol{\theta}|\mathbf{X}, \hat{\mathbf{Z}})$ w.r.t $\delta_{kn\cdot}$ is,

$$\frac{\partial \ell_C}{\partial \delta_{kn\cdot}} = \sum_{c=1}^{C} \hat{z}_{ck}\{\log(1 - x_{cnr}) - [\psi(\delta_{kn\cdot}) - \psi(\alpha_{kn\cdot} + \delta_{kn\cdot})]\}. \tag{8}$$

The lower bound value of the digamma function ($\psi(y) > \log(y - 1/2)$) is used in (7) and (8) to get closed-form solutions at the Maximisation-step of the EM algorithm,

$$\frac{\partial \ell_C}{\partial \alpha_{kn\cdot}} \approx \sum_{c=1}^{C} \hat{z}_{ck} \sum_{n=1}^{N}\sum_{r=1}^{1}\left[\log x_{cnr} - \log \frac{\alpha_{kn\cdot} - 1/2}{\alpha_{kn\cdot} + \delta_{kn\cdot} - 1/2}\right] \tag{9}$$

and

$$\frac{\partial \ell_C}{\partial \delta_{kn\cdot}} \approx \sum_{c=1}^{C} \hat{z}_{ck} \sum_{n=1}^{N}\sum_{r=1}^{1}\left[\log(1 - x_{cnr}) - \log \frac{\delta_{kn\cdot} - 1/2}{\alpha_{kn\cdot} + \delta_{kn\cdot} - 1/2}\right]. \tag{10}$$

Equating (9) and (10) to zero, we get the approximate estimates of $\alpha_{kn\cdot}$ and $\delta_{kn\cdot}$ as,

$$\alpha_{kn\cdot} = 0.5 + \frac{0.5\exp(-y_2)}{\{[\exp(-y_2) - 1][\exp(-y_1) - 1]\} - 1}$$

and

$$\delta_{kn\cdot} = \frac{0.5\exp(-y_2)[\exp(-y_1) - 1]}{\{[\exp(-y_2) - 1][\exp(-y_1) - 1]\} - 1},$$

where $y_1 = (\sum_{c=1}^{C} z_{ck}\log x_{cnr})/(\sum_{c=1}^{C} z_{ck})$ and $y_2 = (\sum_{c=1}^{C} z_{ck}\log(1 - x_{cnr}))/(\sum_{c=1}^{C} z_{ck})$.

Appendix S3

K·R Model The complete data log-likelihood for this model is,

$$\ell_C(\boldsymbol{\tau}, \boldsymbol{\theta}, \mathbf{Z}|\mathbf{X}) = \sum_{c=1}^{C}\sum_{k=1}^{K} z_{ck}\{\log \tau_k + \sum_{n=1}^{N}\sum_{r=1}^{R} \log[\text{Beta}(x_{cnr}; \alpha_{k\cdot r}, \delta_{k\cdot r})]\}.$$

In the Expectation-step of the EM algorithm the $\hat{z}_{ck}$ is calculated given the current parameter estimates. In the Maximisation-step the expected complete data log-likelihood function to be optimized is,

$$\ell_C(\boldsymbol{\tau}, \boldsymbol{\theta}|\mathbf{X}, \hat{\mathbf{Z}}) = \sum_{c=1}^{C}\sum_{k=1}^{K} \hat{z}_{ck}\{\log \tau_k + \sum_{n=1}^{N}\sum_{r=1}^{R}[(\alpha_{k\cdot r} - 1)\log x_{cnr} + (\delta_{k\cdot r} - 1)\log(1 - x_{cnr}) - \log \text{B}(\alpha_{k\cdot r}, \delta_{k\cdot r})]\}. \tag{11}$$

Differentiating (11) w.r.t $\alpha_{k\cdot r}$ yields,

$$\frac{\partial \ell_C}{\partial \alpha_{k\cdot r}} = \sum_{c=1}^{C} \hat{z}_{ck}\{\log x_{cnr} - [\psi(\alpha_{k\cdot r}) - \psi(\alpha_{k\cdot r} + \delta_{k\cdot r})]\} \tag{12}$$

where ψ is the digamma function.

Similarly, the derivative of $\ell_C(\boldsymbol{\tau}, \boldsymbol{\theta}|\mathbf{X}, \hat{\mathbf{Z}})$ w.r.t $\delta_{k\cdot r}$ is,

$$\frac{\partial \ell_C}{\partial \delta_{k\cdot r}} = \sum_{c=1}^{C} \hat{z}_{ck}\{\log(1 - x_{cnr}) - [\psi(\delta_{k\cdot r}) - \psi(\alpha_{k\cdot r} + \delta_{k\cdot r})]\}. \tag{13}$$

The lower bound value of the digamma function ($\psi(y) > \log(y - 1/2)$) is used in (12) and (13) to get closed-form solutions at the Maximisation-step of the EM algorithm,

$$\frac{\partial \ell_C}{\partial \alpha_{k\cdot r}} \approx \sum_{c=1}^{C} \hat{z}_{ck} \sum_{n=1}^{N}\sum_{r=1}^{1}\left[\log x_{cnr} - \log \frac{\alpha_{k\cdot r} - 1/2}{\alpha_{k\cdot r} + \delta_{k\cdot r} - 1/2}\right] \tag{14}$$

and

$$\frac{\partial \ell_C}{\partial \delta_{k\cdot r}} \approx \sum_{c=1}^{C} \hat{z}_{ck} \sum_{n=1}^{N}\sum_{r=1}^{1}\left[\log(1 - x_{cnr}) - \log \frac{\delta_{k\cdot r} - 1/2}{\alpha_{k\cdot r} + \delta_{k\cdot r} - 1/2}\right]. \tag{15}$$

Equating (14) and (15) to zero, we get the approximate estimates of α_{knr} and δ_{knr} as,

$$\alpha_{k\cdot r} = 0.5 + \frac{0.5\exp(-y_2)}{\{[\exp(-y_2) - 1][\exp(-y_1) - 1]\} - 1}$$

and

$$\delta_{k\cdot r} = \frac{0.5\exp(-y_2)[\exp(-y_1) - 1]}{\{[\exp(-y_2) - 1][\exp(-y_1) - 1]\} - 1},$$

where $y_1 = (\sum_{c=1}^{C} z_{ck}\log x_{cnr})/(N\sum_{c=1}^{C} z_{ck})$ and $y_2 = (\sum_{c=1}^{C} z_{ck}\log(1 - x_{cnr}))/(N\sum_{c=1}^{C} z_{ck})$.

Appendix S4

Table 1: Beta distributions' parameter estimates for sample type A in a simulated dataset under the K·· model

Clusters	$\hat{\alpha}$	$\hat{\delta}$	Mean	Std. deviation
1	4.161	3.129	0.571	0.336
2	1.396	14.092	0.090	0.077
3	13.761	1.371	0.909	0.273

Table 2: Beta distributions' parameter estimates for sample type A in a simulated dataset under the K·R model

.

(a) Sample A

Clusters	$\hat{\alpha}$	$\hat{\delta}$	Mean	Std. deviation
1	1.391	14.024	0.090	0.077
2	13.918	1.386	0.909	0.273
3	1.384	13.954	0.090	0.078
4	4.168	3.137	0.571	0.335
5	4.157	3.134	0.570	0.335
6	13.832	1.383	0.909	0.274
7	13.987	1.398	0.909	0.274
8	4.155	3.134	0.570	0.335
9	1.395	14.088	0.090	0.077

(b) Sample B

Clusters	$\hat{\alpha}$	$\hat{\delta}$	Mean	Std. deviation
1	13.908	1.383	0.091	0.273
2	1.393	14.060	0.090	0.077
3	4.186	3.154	0.570	0.335
4	1.411	14.207	0.090	0.077
5	13.909	1.391	0.909	0.274
6	4.156	3.128	0.571	0.336
7	13.857	1.384	0.909	0.274
8	4.150	3.124	0.571	0.336
9	1.385	13.981	0.090	0.078

Table 3: Beta distributions' parameter estimates for benign sample type in the PCa dataset under the KN· model.

(a) Patient 1

Clusters	$\hat{\alpha}$	$\hat{\delta}$	Mean	Std. deviation
1	13.774	2.205	0.862	0.084
2	1.491	12.454	0.107	0.080
3	3.970	2.965	0.572	0.176

(c) Patient 2

Clusters	$\hat{\alpha}$	$\hat{\delta}$	Mean	Std. deviation
1	21.434	2.871	0.882	0.064
2	2.166	18.166	0.107	0.067
3	4.111	2.980	0.580	0.174

(b) Patient 3

Clusters	$\hat{\alpha}$	$\hat{\delta}$	Mean	Std. deviation
1	20.158	2.624	0.885	0.065
2	2.183	28.896	.070	0.045
3	3.618	3.023	0.545	0.180

(d) Patient 4

Clusters	$\hat{\alpha}$	$\hat{\delta}$	Mean	Std. deviation
1	26.825	2.644	0.910	0.052
2	2.462	30.940	0.074	0.045
3	3.338	2.237	0.599	0.191

Appendix S5

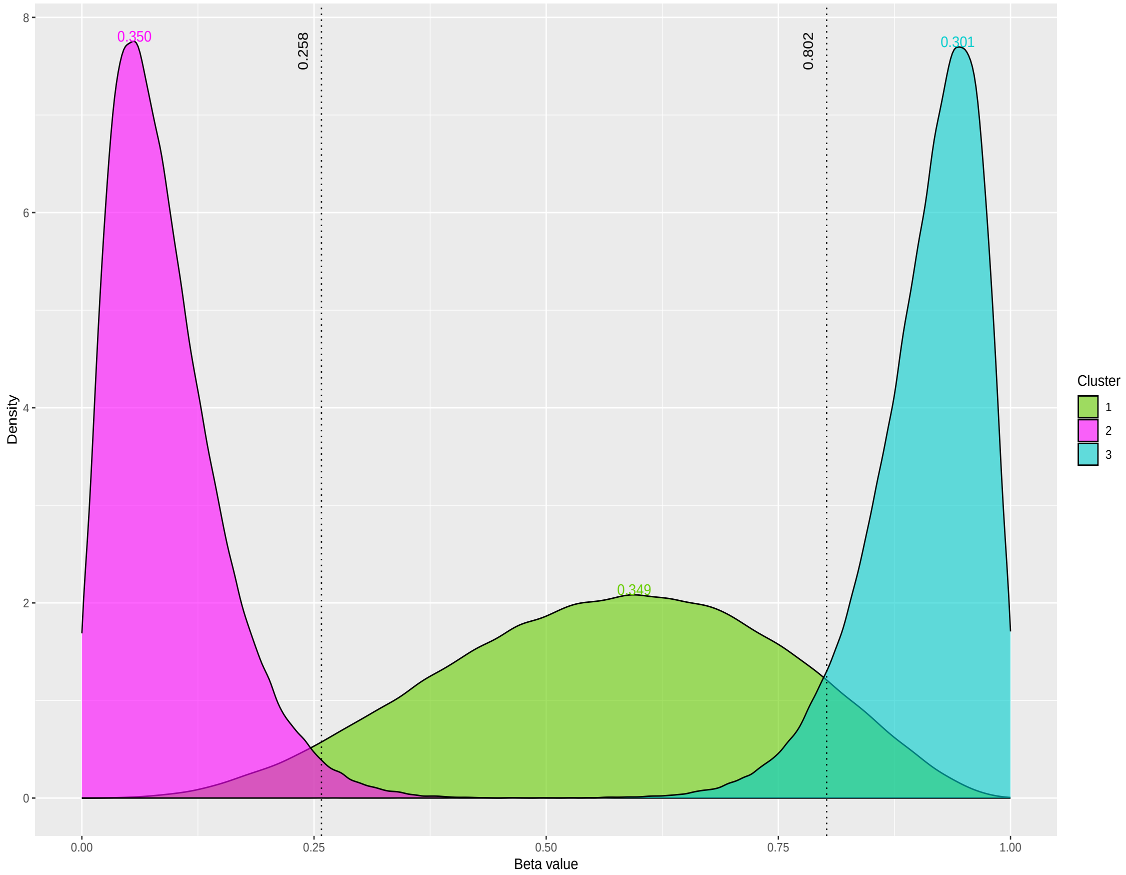

Figure 1: Kernel density estimates under the K·· model fitted to data from sample type A in the simulated dataset. The thresholds are 0.258 and 0.802. The estimated mixing proportions are displayed.

Appendix S6

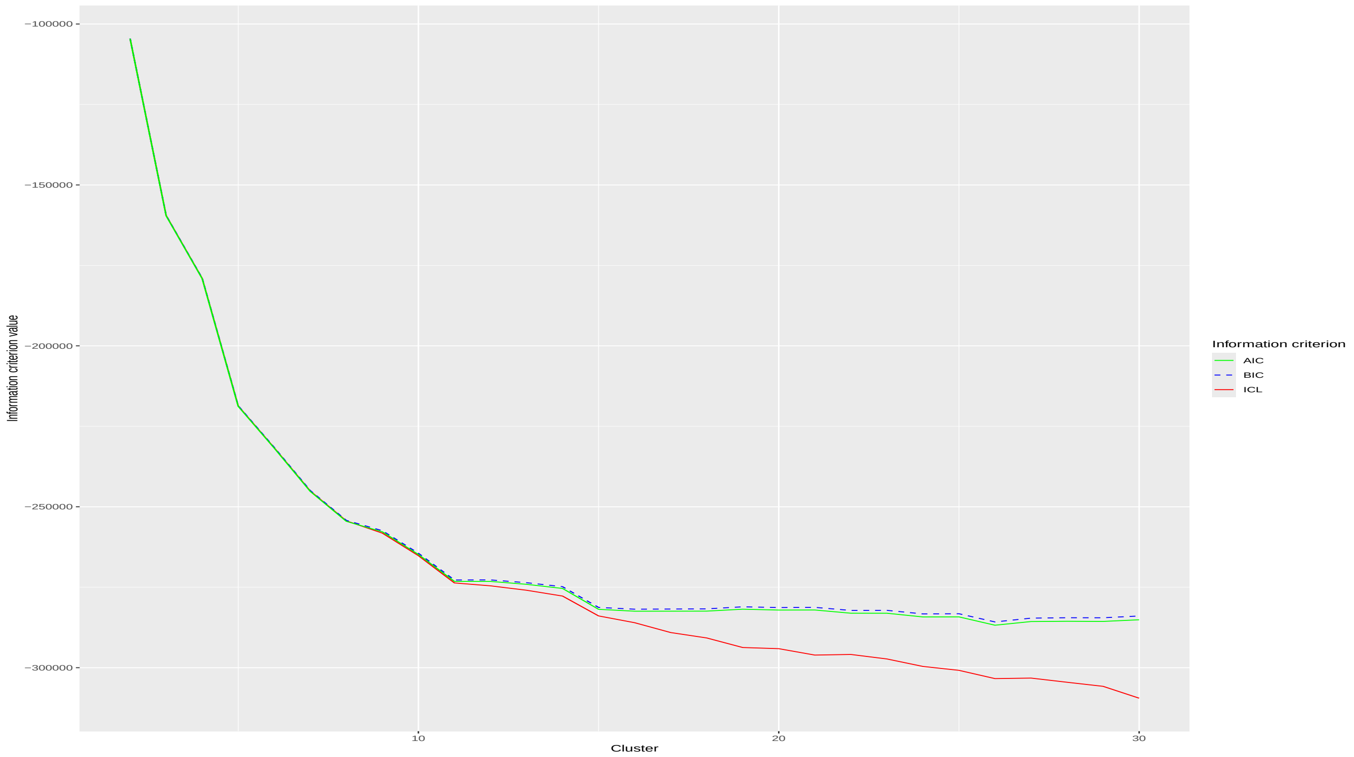

Figure 2: The AIC, BIC and ICL information criteria for different numbers of clusters, K, for the simulated datasets.

Appendix S7

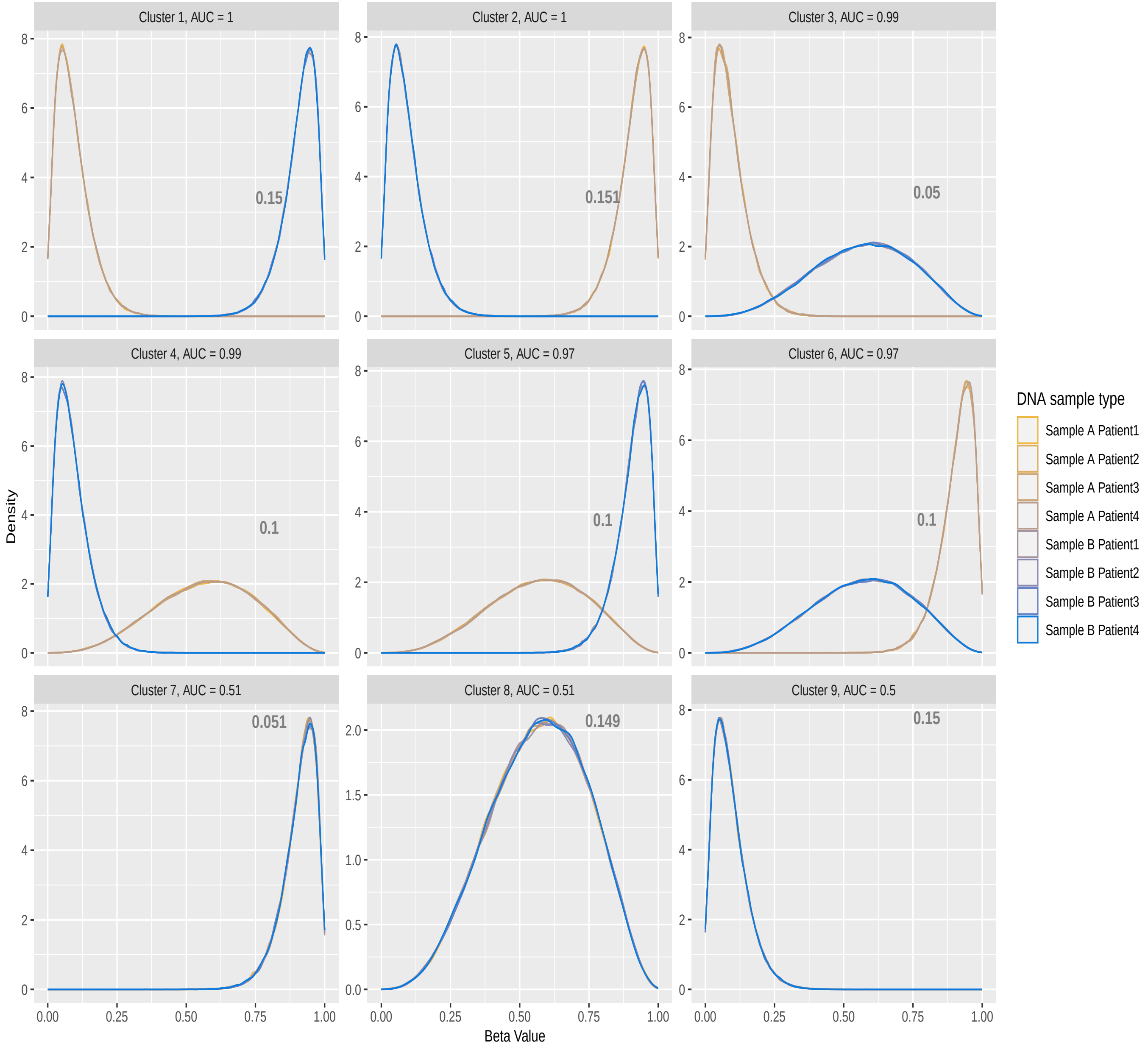

Figure 3: Kernel density estimates under the clustering solution of the K·R model fitted to DNA samples from sample A and sample B from a simulated dataset. The estimated mixing proportions are displayed in the relevant panel.

Appendix S8

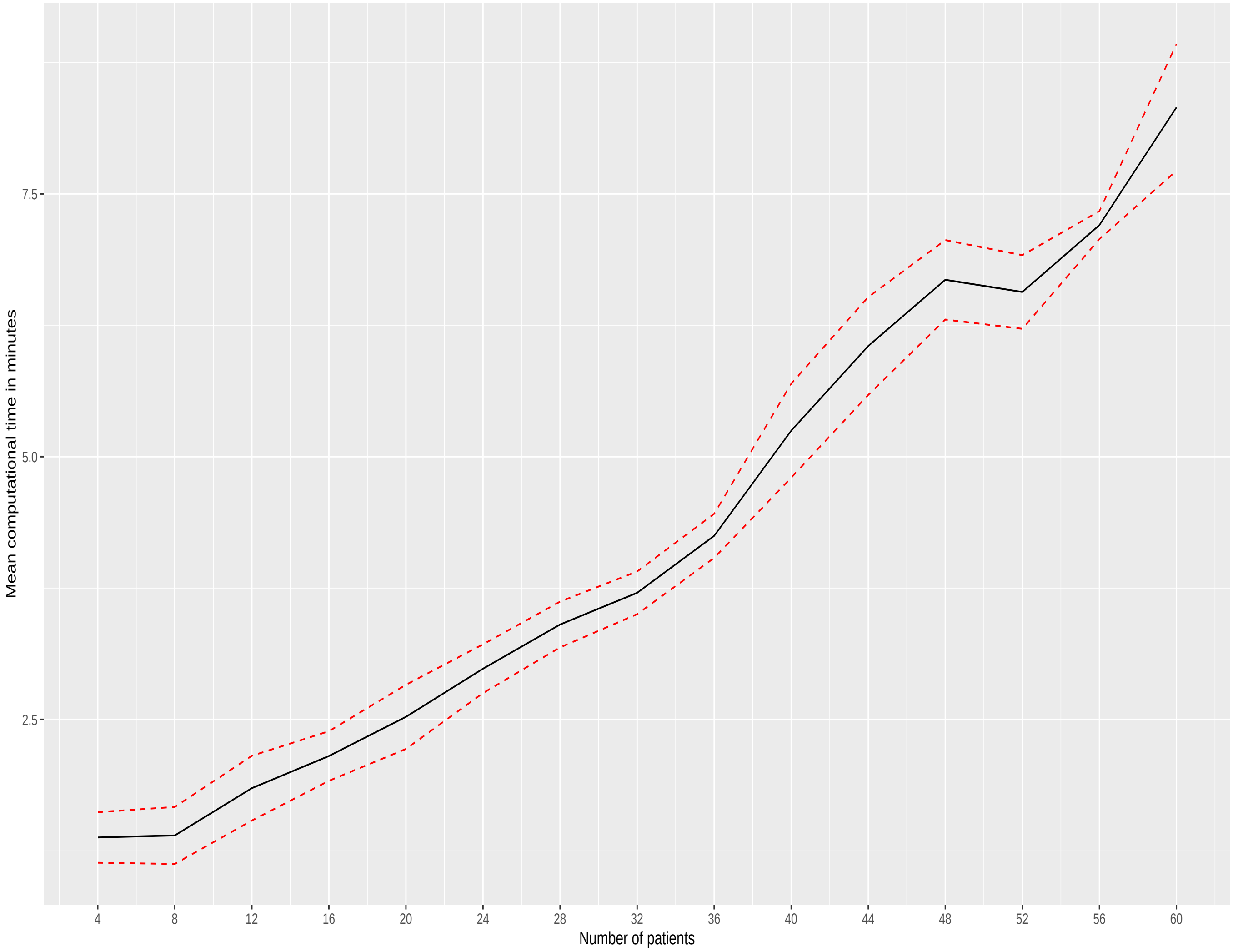

Figure 4: Mean computational time for fitting the K·R model, with 95% confidence intervals, as the number of patients N is increased. The computational times for the K·· and KN· models show a similar trend, with elapsed times ranging from 0.33 to 2.5 minutes for the former and 0.47 to 4 minutes for the latter. As the complexity of the algorithm with respect to N is proportional to N, as the number of patients increases the computational cost scales linearly.

Appendix S9

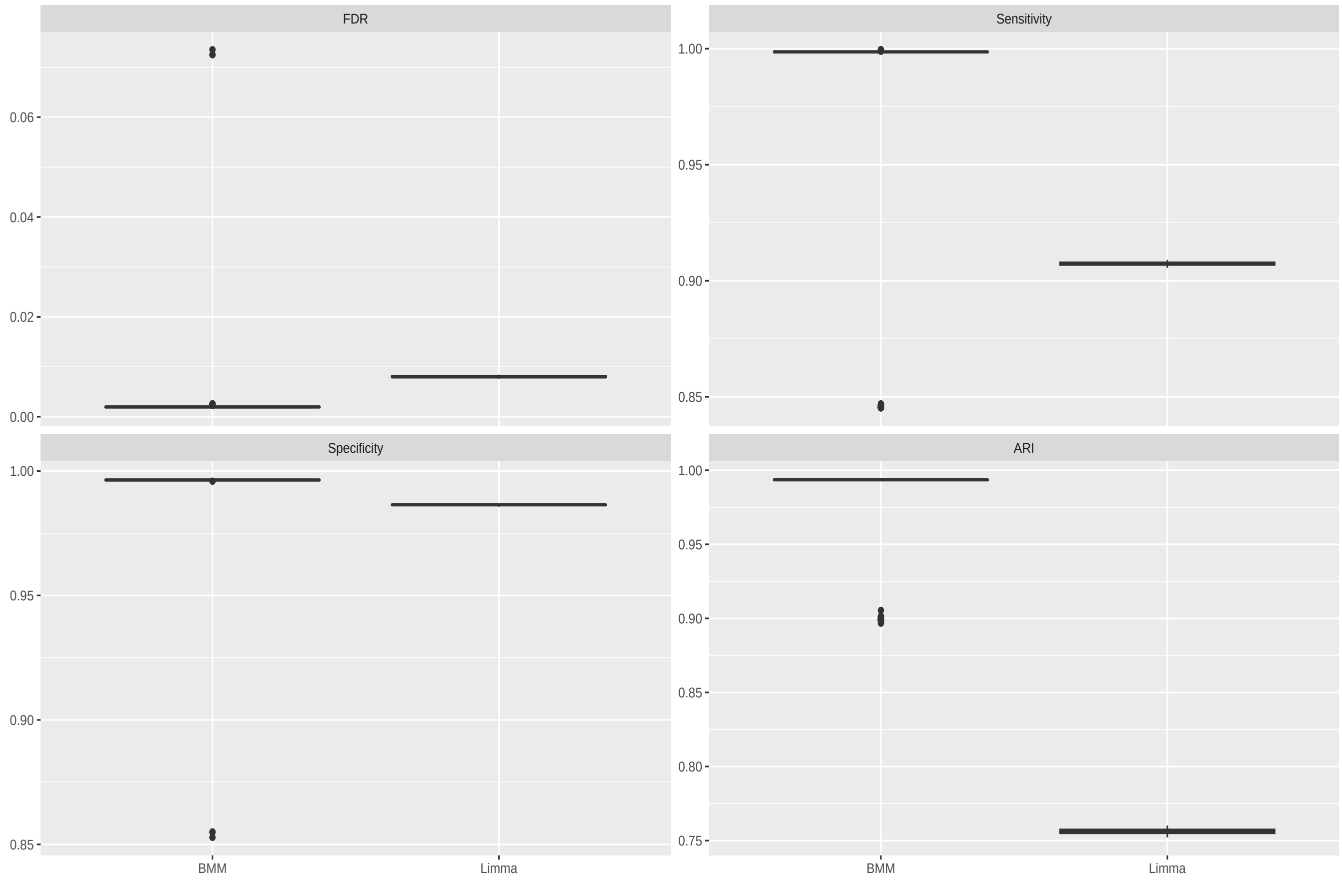

Figure 5: Boxplot displaying the FDR, sensitivity, specificity and ARI values from the BMM and Limma methods when applied to the simulated data from a mixture of beta distributions.

Appendix S10

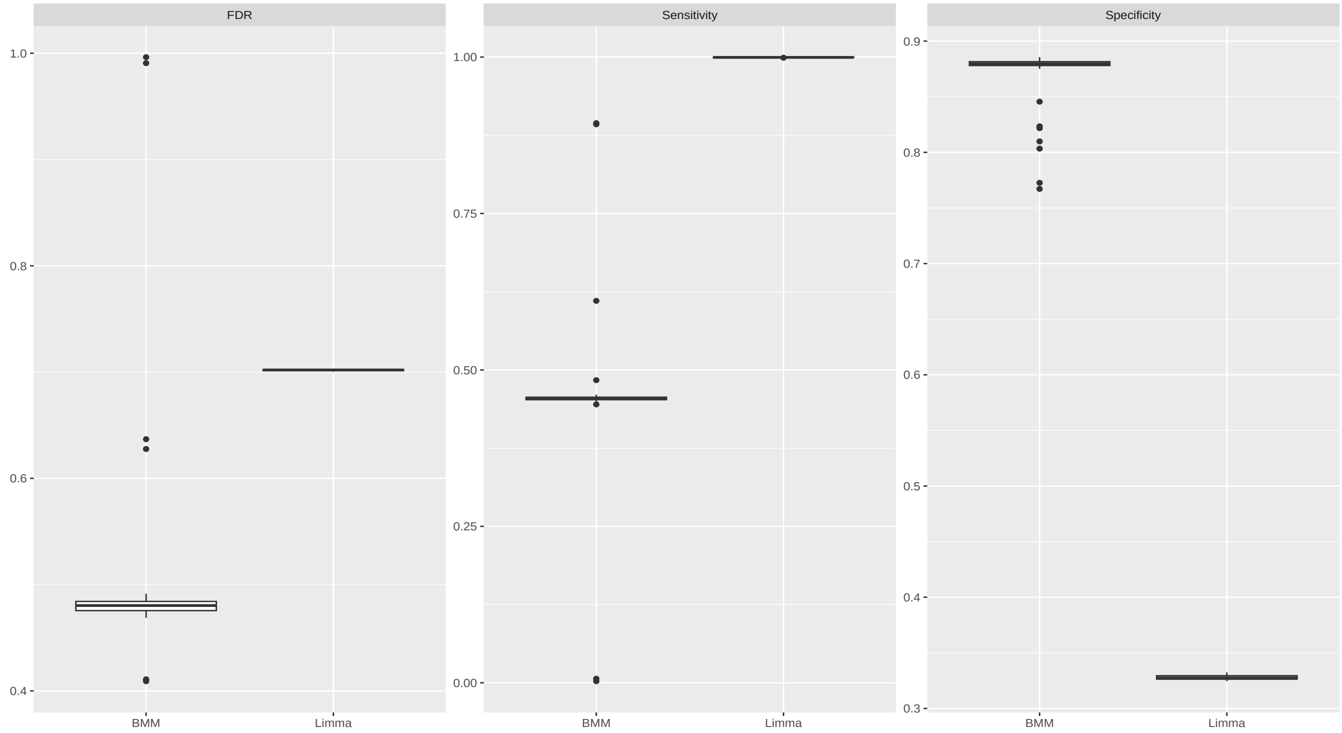

Figure 6: Boxplot showing the FDR, sensitivity and specificity values from the BMM and Limma methods applied to the simulated datasets generated from scaled t-distribution with 8 degrees of freedom to assess the impact of model misspecification.

Appendix S11

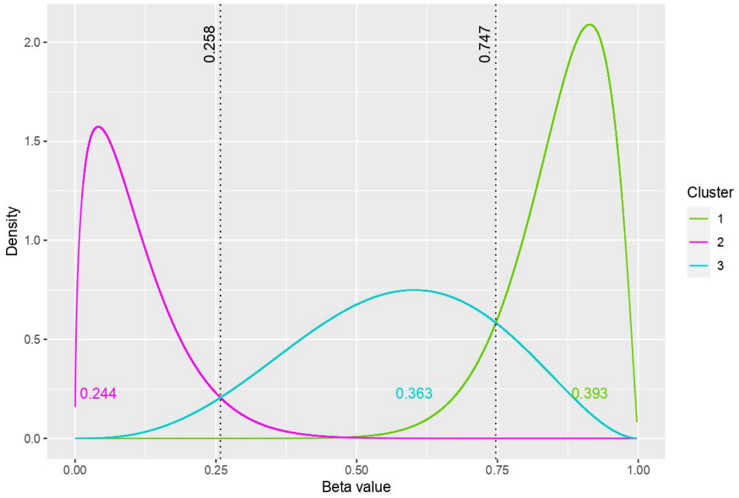

Figure 7: Fitted density estimates under the clustering solution of the KN· model fitted to the benign sample collected from patient 1 in the prostate cancer dataset. The threshold points are illustrated in the graph as 0.258 and 0.747.

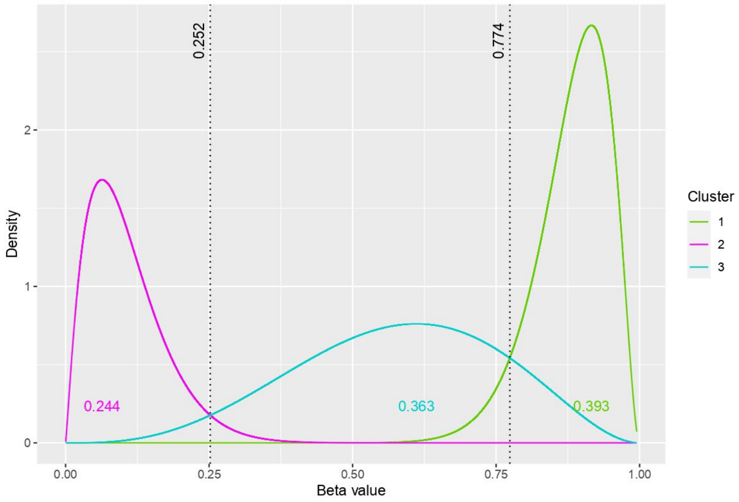

Figure 8: Fitted density estimates under the clustering solution of the KN· model fitted to the benign sample collected from patient 2 in the prostate cancer dataset. The threshold points are illustrated in the graph as 0.252 and 0.774.

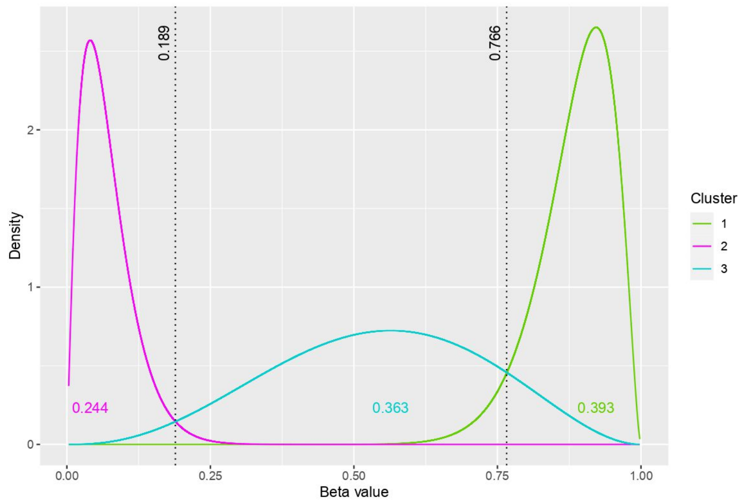

Figure 9: Fitted density estimates under the clustering solution of the KN· model fitted to the benign sample collected from patient 3 in the prostate cancer dataset. The threshold points are illustrated in the graph as 0.189 and 0.766.

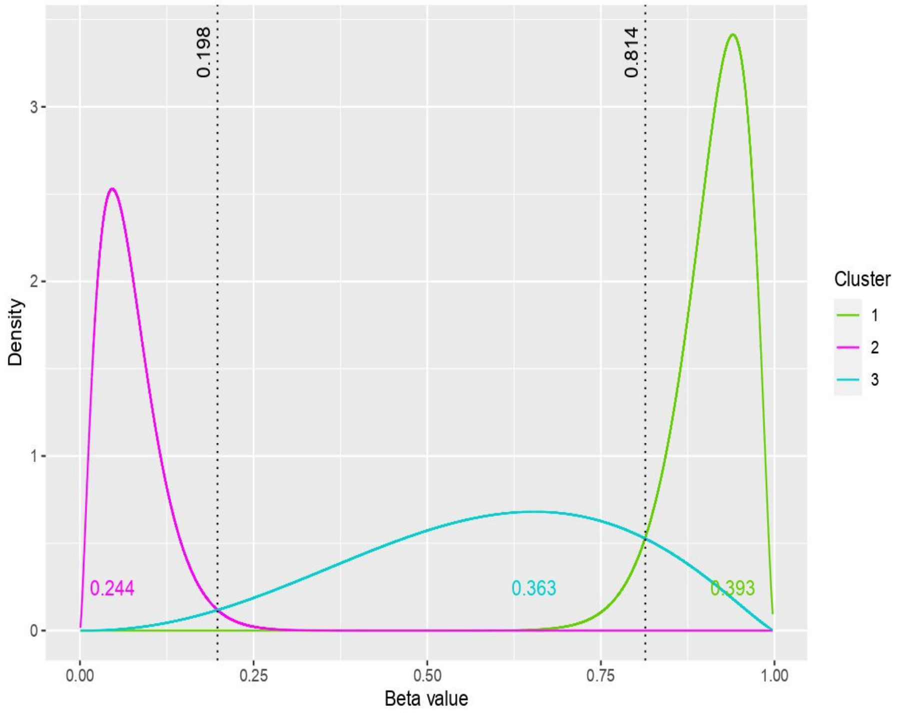

Figure 10: Fitted density estimates under the clustering solution of the KN· model fitted to the benign sample collected from patient 4 in the prostate cancer dataset. The threshold points are illustrated in the graph as 0.198 and 0.814.

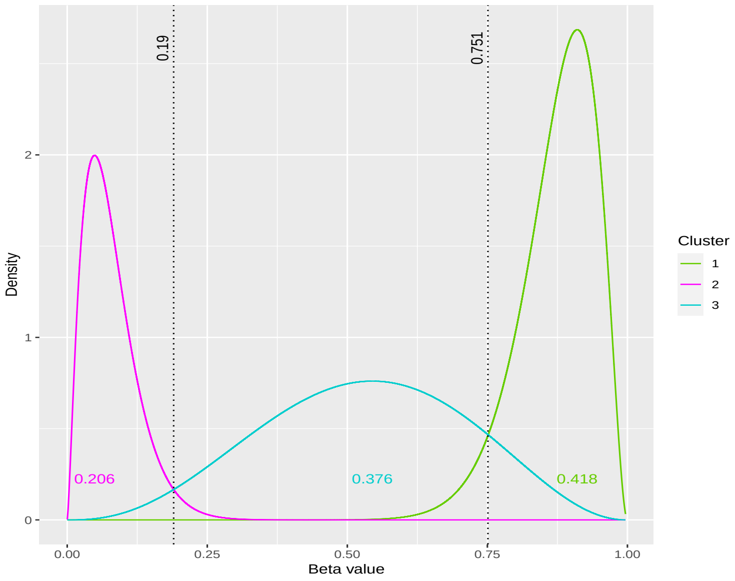

Figure 11: Fitted density estimates under the clustering solution of the KN· model fitted to the tumour sample collected from patient 1 in the prostate cancer dataset. The threshold points are illustrated in the graph as 0.19 and 0.751.

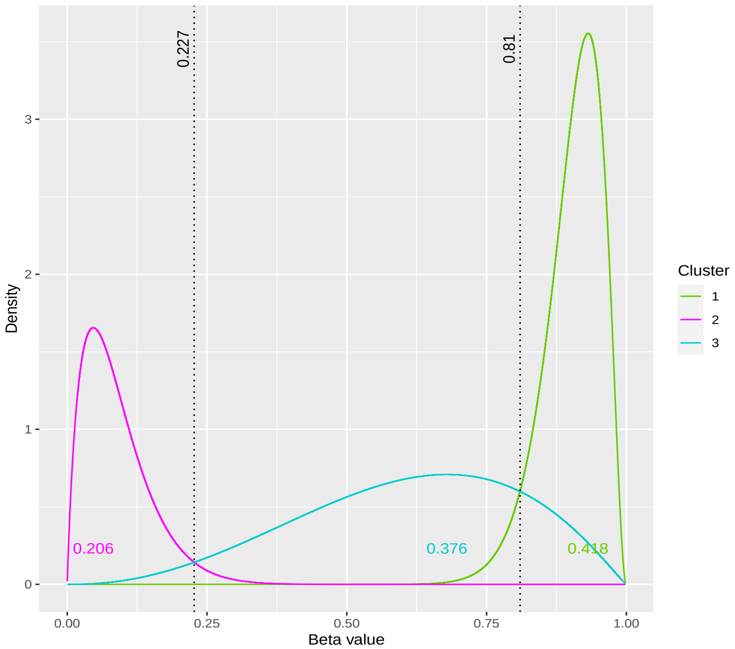

Figure 12: Fitted density estimates under the clustering solution of the KN· model fitted to the tumour sample collected from patient 2 in the prostate cancer dataset. The threshold points are illustrated in the graph as 0.227 and 0.81.

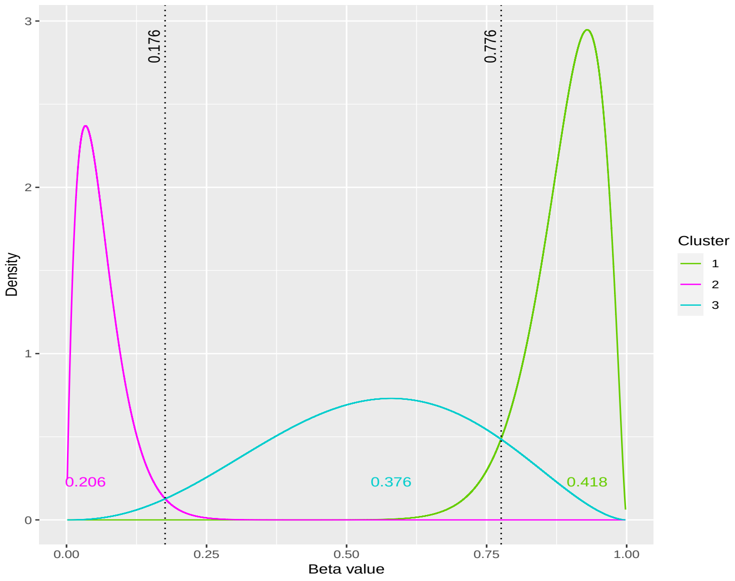

Figure 13: Fitted density estimates under the clustering solution of the KN· model fitted to the tumour sample collected from patient 3 in the prostate cancer dataset. The threshold points are illustrated in the graph as 0.176 and 0.776.

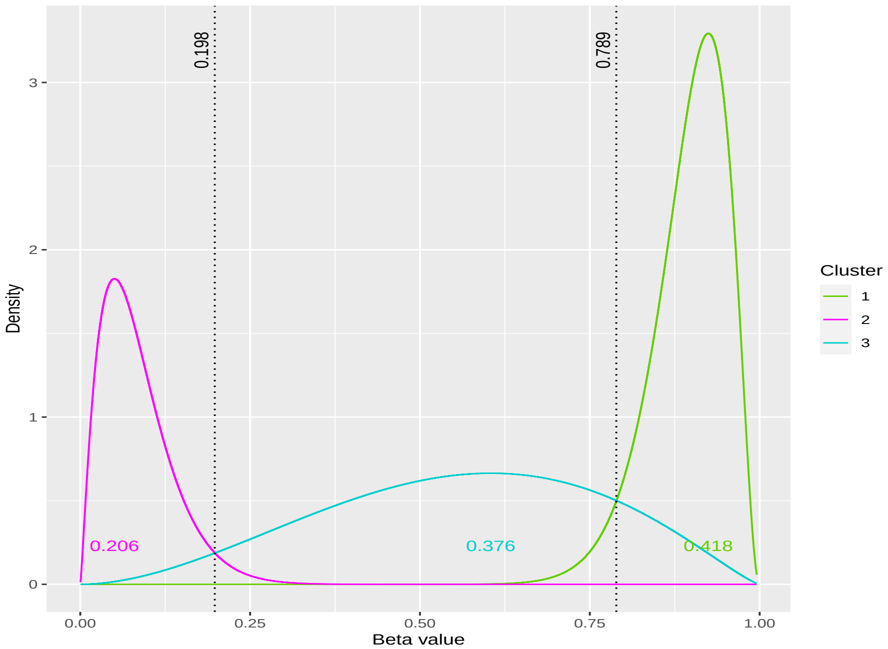

Figure 14: Fitted density estimates under the clustering solution of the KN· model fitted to the tumour sample collected from patient 4 in the prostate cancer dataset. The threshold points are illustrated in the graph as 0.198 and 0.789.

Appendix S12

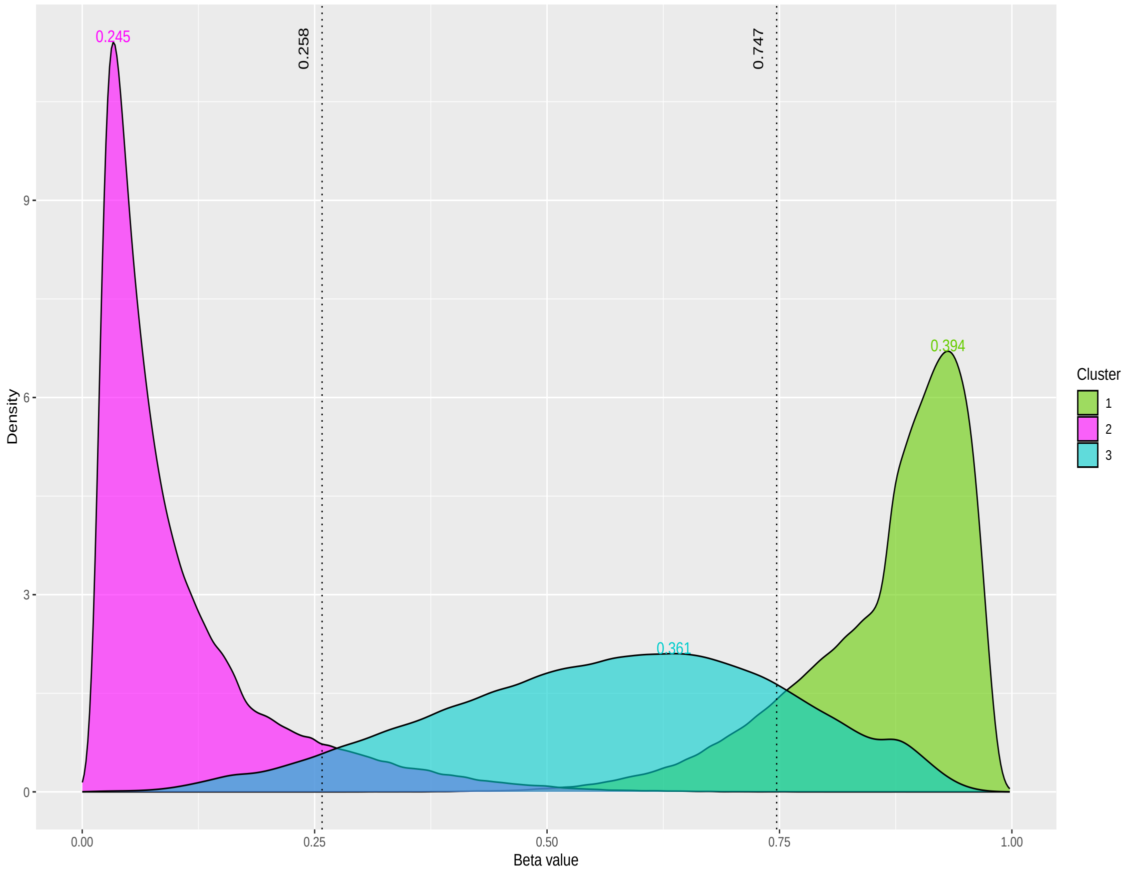

Figure 15: Kernel density estimates under the clustering solution of the KN· model fitted to DNA methylation data from the benign sample collected from patient 1 in the prostate cancer dataset. The thresholds are illustrated along with the estimated mixing proportions.

Appendix S13

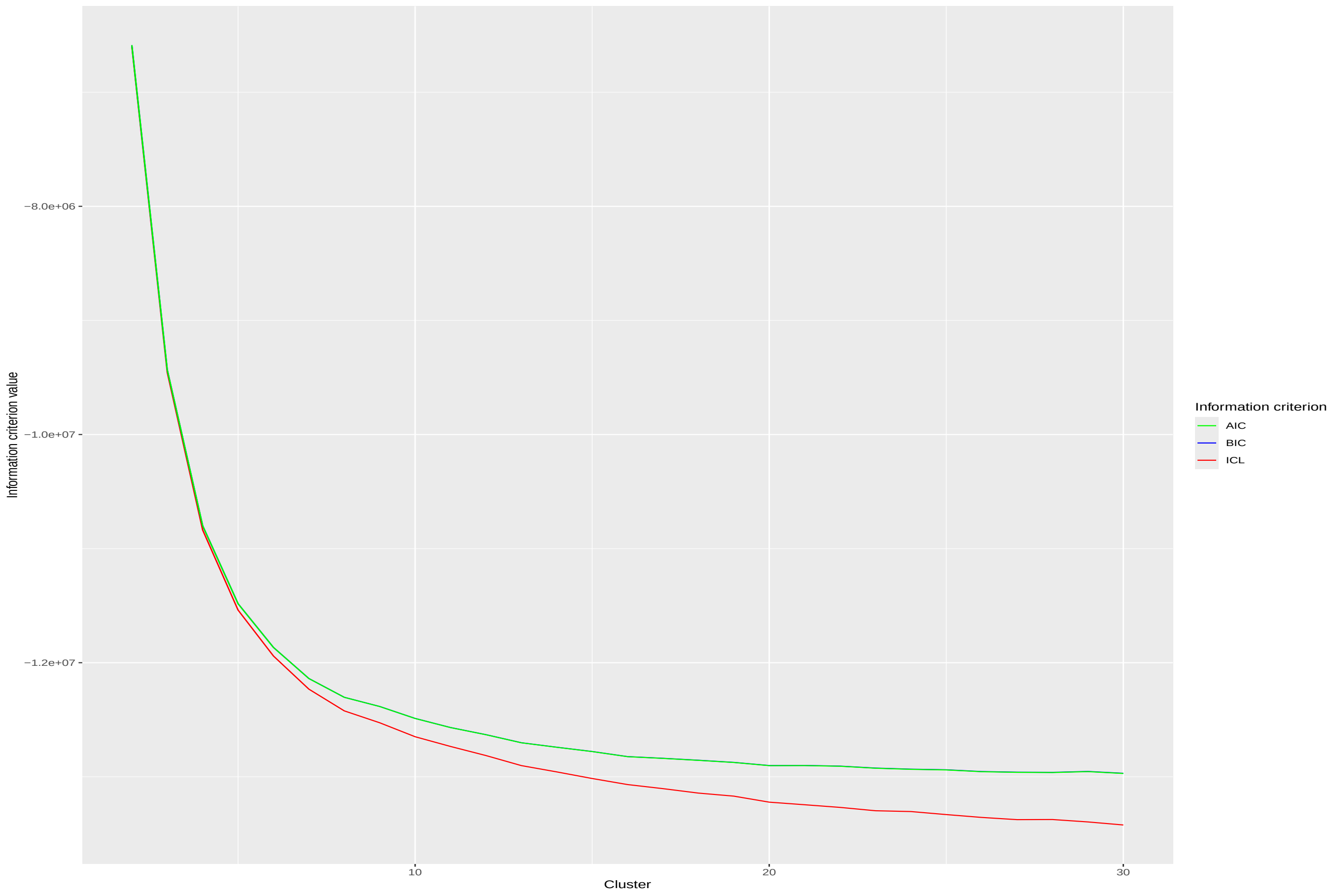

Figure 16: The AIC, BIC and ICL information criteria for different numbers of clusters, K, for the PCa dataset.

Appendix S14

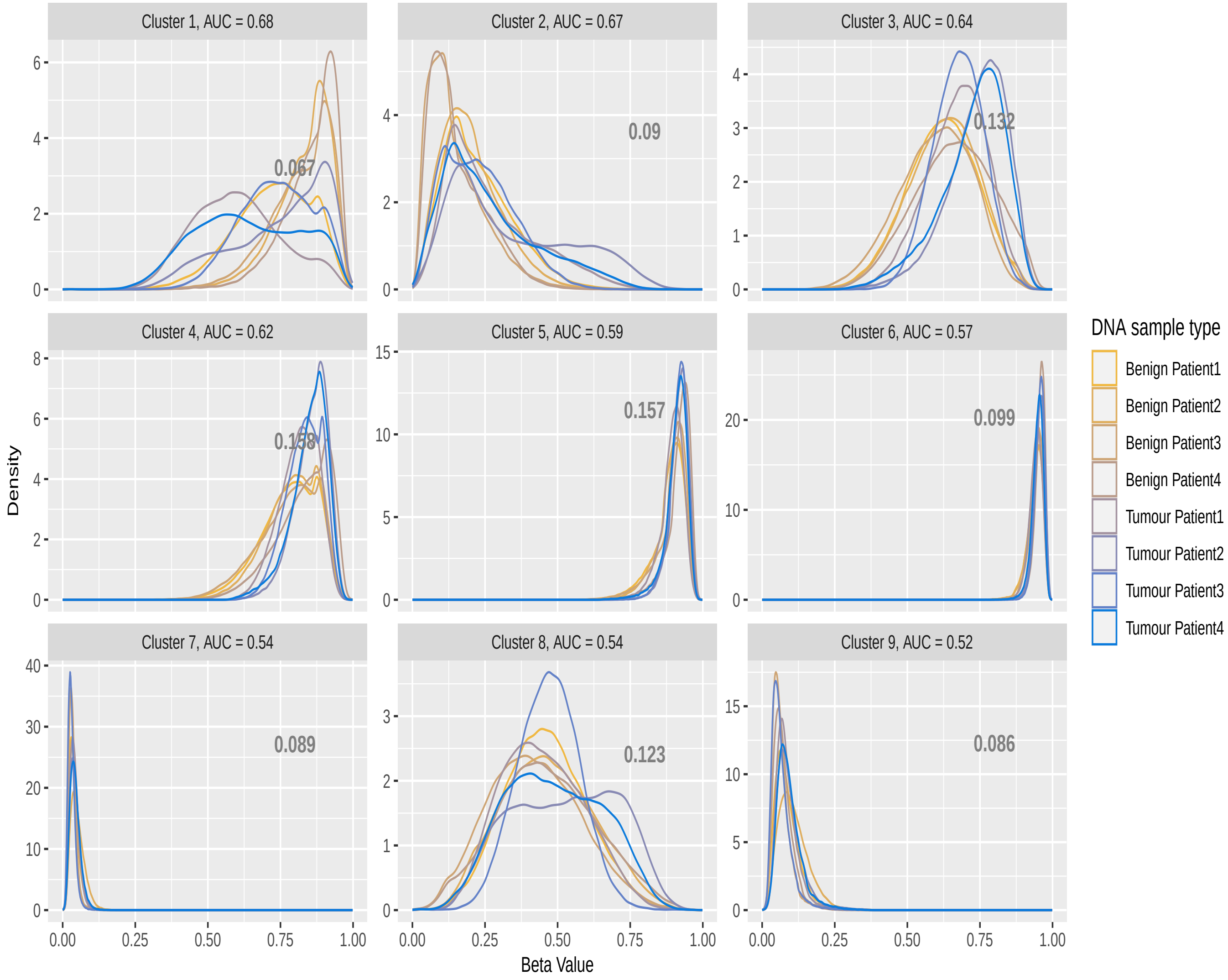

Figure 17: Kernel density estimates under the clustering solution of the K·R model fitted to the DNA methylation data from benign and tumour prostate cancer samples. The estimated mixing proportions are displayed in the relevant panel.

Appendix S15

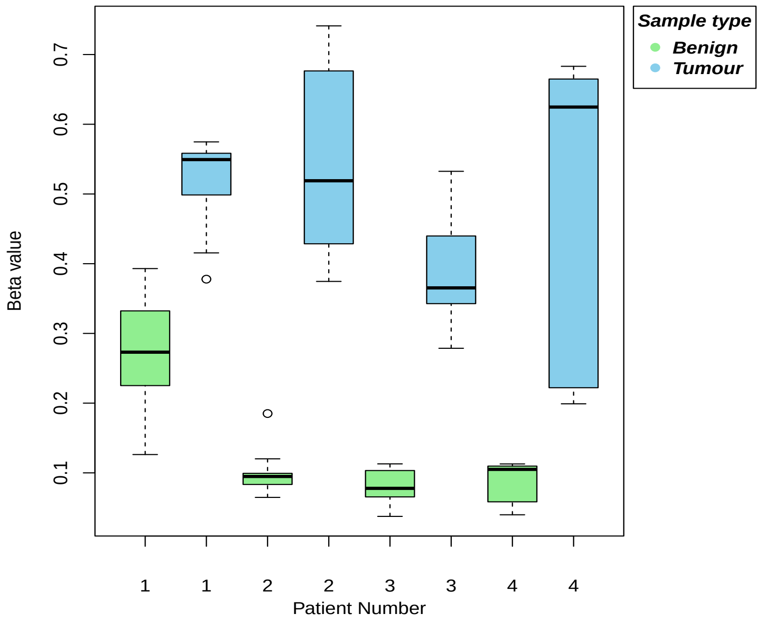

Figure 18: Methlyation levels of the differentially methylated CpG sites related to the RARB genes in the benign and tumour sample types.

Appendix S16

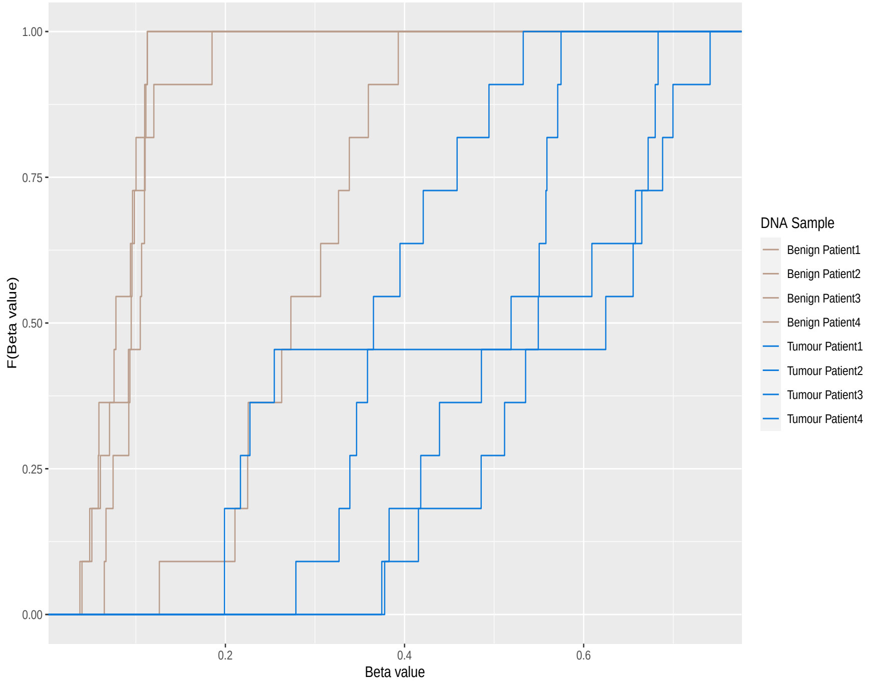

Figure 19: ECDFs for the DMCs related to the RARB genes for all patients and sample types.

Appendix S17

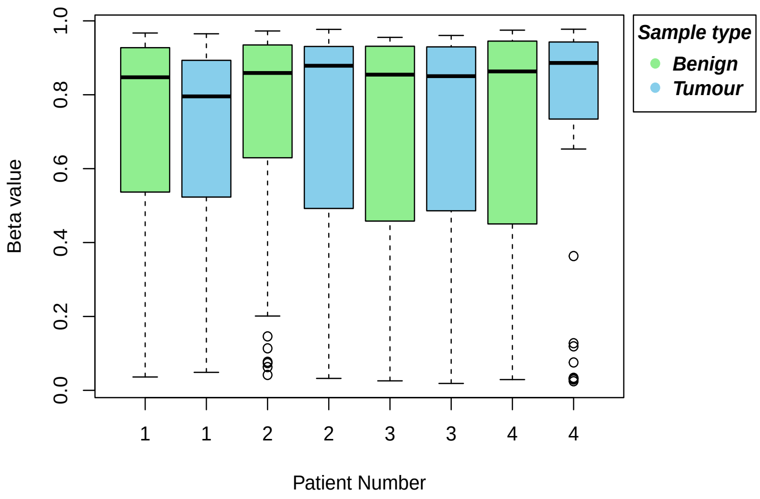

Figure 20: Methylation levels of the differentially methylated CpG sites in clusters 3-9 related to the AKT1 gene for all patients and sample types.

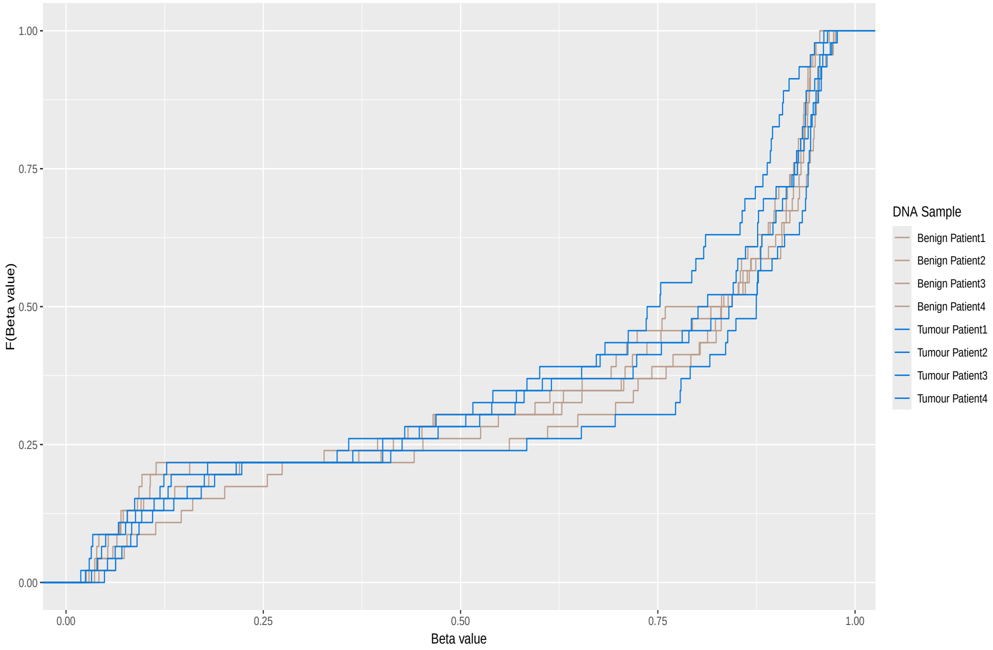

Figure 21: ECDFs for the CpG sites in clusters 3-9 related to the AKT1 gene for all patients and sample types.

Appendix S18

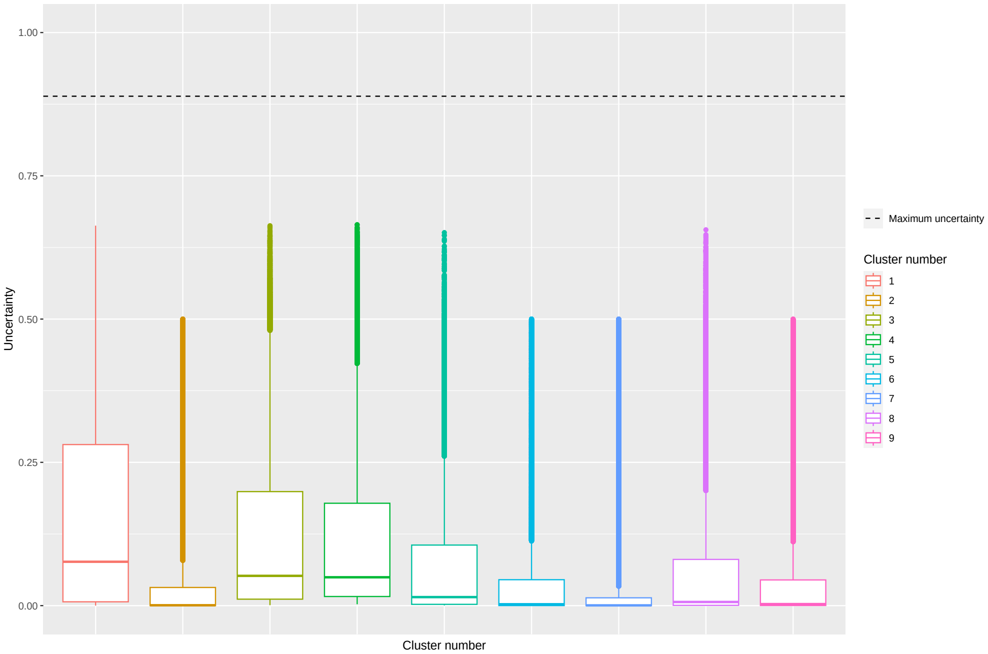

Figure 22: Clustering uncertainties for CpG sites in the PCa data.
