# Supplementary material for: A novel family of beta mixture models for the differential analysis of DNA methylation data: An application to prostate cancer
Source: PLoS One. 2024 Dec 11;19(12):e0314014. doi: 10.1371/journal.pone.0314014 (PMC11633993; doi:10.1371/journal.pone.0314014)
Supplement: S2 File — Supporting Information (Appendix S19) contains analysis of BMMs applied to the additional esophageal squamous cell carcinoma dataset. (PDF) [file pone.0314014.s002.pdf]

Supporting Information 2 for ‘A novel family of beta mixture models for the differential analysis of DNA methylation data: an application to prostate cancer’ data by Majumdar et al.

Appendix S19

Esophageal squamous cell carcinoma data

Esophageal squamous cell carcinoma (ESCC) is a subtype of esophageal cancer characterized by aberrant DNA methylation. A study was conducted to investigate abnormal genes in ESCC, and DNA samples were collected from 15 patients’ benign and tumour tissues (1). Paired samples from 4 randomly selected patients were considered. Approximately 1.34 % of the methylation values were 0; given this low prevalence, here 0 values were substituted with the minimum observed *beta* value. The ESCC dataset contained observed *beta* values for $C = 481,315$ CpG sites from each of $R = 2$ DNA sample types collected from each of $N = 4$ patients. These data were accessed from GEO repository(GSE121931) on 23rd of February, 2022 for research purposes. The authors had no access to information that could identify individual participants.

Estimating methylation state thresholds

The methylation states of each CpG site and the threshold points between these states are to be inferred. The K·· and KN· models are used to achieve this objective by clustering the CpG sites from the benign sample into 3 methylation states, allowing objective inference of the thresholds. The KN· model was selected as the optimal model by BIC and the fitted density estimates of the clustering solution for patient 1 are displayed in Figure 1. As the KN· model estimates different parameters for each patient, different pairs of thresholds are calculated for each patient. The methylation state thresholds for patient 1 are inferred to be 0.334 and 0.79 under the KN· model. A summary of the parameter estimates under the KN· model is presented in Table 1.

Identifying DMCs in the ESCC data

The CpG sites that are differentially methylated between the benign and tumour samples are identified by fitting the K·R model with biologically motivated $K = 9$ clusters. The fitted densities are shown in Figure 2. A summary of the parameter estimates under the K·R model is presented in Table 2.

The maximum possible uncertainty when clustering the CpG sites into K clusters is $1 - 1/K = 8/9$. Figure 3 illustrates the clustering uncertainties for all CpG sites and demonstrates that there is low uncertainty in the CpG site’s cluster memberships under the K·R model. We subsequently utilized the AUC and WD metrics to quantify the dissimilarity among the cumulative distributions within each cluster. The clusters are illustrated in the descending order of their degree of differential methylation, based on decreasing AUC and, in the case of ties, WD values. These metrics and the parameter estimates suggest clusters

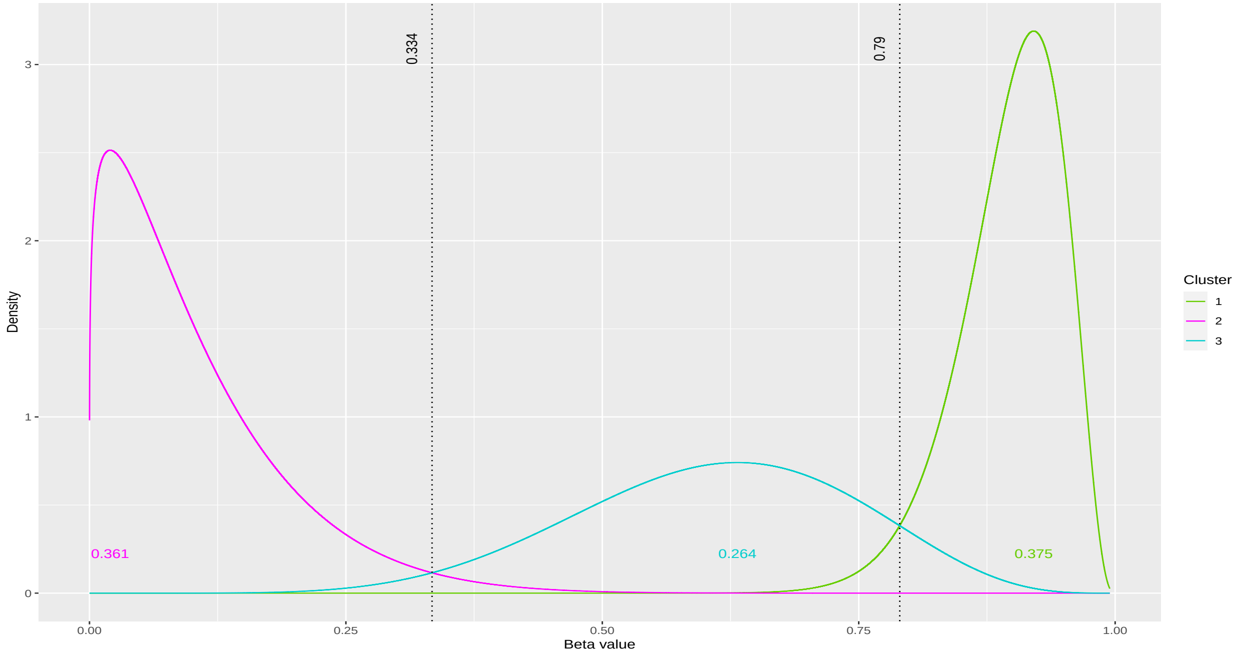

Figure 1: Fitted density estimates under the clustering solution of the KN· model for DNA methylation data from the benign sample collected from patient 1 in the ESCC dataset. The methylation state thresholds are illustrated by the black dotted lines along with the estimated mixing proportions.

Table 1: Beta distributions' parameter estimates for benign samples in the ESCC dataset under the KN· model.

(a) Patient 1

Clusters	$\hat{\alpha}$	$\hat{\delta}$	Mean	Std. deviation
1	31.640	3.642	0.897	0.051
2	1.193	10.385	0.103	0.086
3	7.369	4.713	0.610	0.135

(b) Patient 3

Clusters	$\hat{\alpha}$	$\hat{\delta}$	Mean	Std. deviation
1	28.449	3.880	0.880	0.056
2	1.278	10.7	0.107	0.086
3	7.509	5.005	0.600	0.133

(c) Patient 2

Clusters	$\hat{\alpha}$	$\hat{\delta}$	Mean	Std. deviation
1	31.532	3.632	0.897	0.051
2	1.162	10.587	0.099	0.084
3	6.600	4.274	0.607	0.142

(d) Patient 4

Clusters	$\hat{\alpha}$	$\hat{\delta}$	Mean	Std. deviation
1	29.519	3.986	0.881	0.055
2	1.281	9.552	0.118	0.094
3	7.509	4.948	0.593	0.135

1 and 2 to be the most differentially methylated clusters thus identifying 104,930 CpG sites as being the most differentially methylated CpG sites. Detailed AUC and WD metric values for each individual cluster can be found in Table 3.

We conducted gene ontology analysis (2) on the differentially methylated clusters, namely cluster 1 and cluster 2. In cluster 1, a total of 439 significant biological processes were identified (FDR adjusted p-value < 0.05), while cluster 2 revealed 385 significant biological

Table 2: Beta distributions' parameter estimates for the ESCC dataset under the K·R model.

.

(a) Benign samples

Clusters	$\hat{\alpha}$	$\hat{\delta}$	Mean	Std. deviation
1	12.508	18.222	0.407	0.087
2	1.968	9.562	0.171	0.106
3	72.289	10.548	0.873	0.036
4	114.017	4.712	0.960	0.018
5	31.205	19.781	0.612	0.068
6	170.783	14.678	0.921	0.020
7	41.830	12.567	0.769	0.057
8	8.884	94.511	0.086	0.027
9	1.496	63.762	0.023	0.018

(b) Tumour samples

Clusters	$\hat{\alpha}$	$\hat{\delta}$	Mean	Std. deviation
1	3.230	3.181	0.504	0.184
2	1.179	2.897	0.289	0.201
3	16.145	3.174	0.836	0.082
4	30.385	1.876	0.942	0.041
5	5.679	3.176	0.641	0.153
6	82.000	7.538	0.916	0.029
7	8.581	2.944	0.745	0.123
8	7.922	81.721	0.088	0.030
9	1.421	56.256	0.025	0.020

Table 3: The AUC and WD metrics calculated across each cluster for the ESCC dataset.

	Cluster								
	1	2	3	4	5	6	7	8	9
AUC	0.691	0.679	0.650	0.611	0.588	0.547	0.535	0.529	0.524
WD	0.114	0.119	0.043	0.020	0.076	0.008	0.054	0.003	0.002

processes. Additionally, the DMCs within cluster 1 exhibited relevance to 37 significant KEGG pathways, while those within cluster 2 were associated with 26 significant KEGG pathways. The K·R model identified DMCs related to genes implicated in esophageal squamous cell carcinogenesis. For example, the expression of the GPX3 gene has been shown to be downregulated in ESCC when compared with normal esophageal mucosa (3). The promoter methylation results in the silencing of the GPX3 genes in ESCC. The ECDF plot in Figure 4 illustrates hypermethylation of the identified DMCs related to the GPX3 gene in the tumour samples.

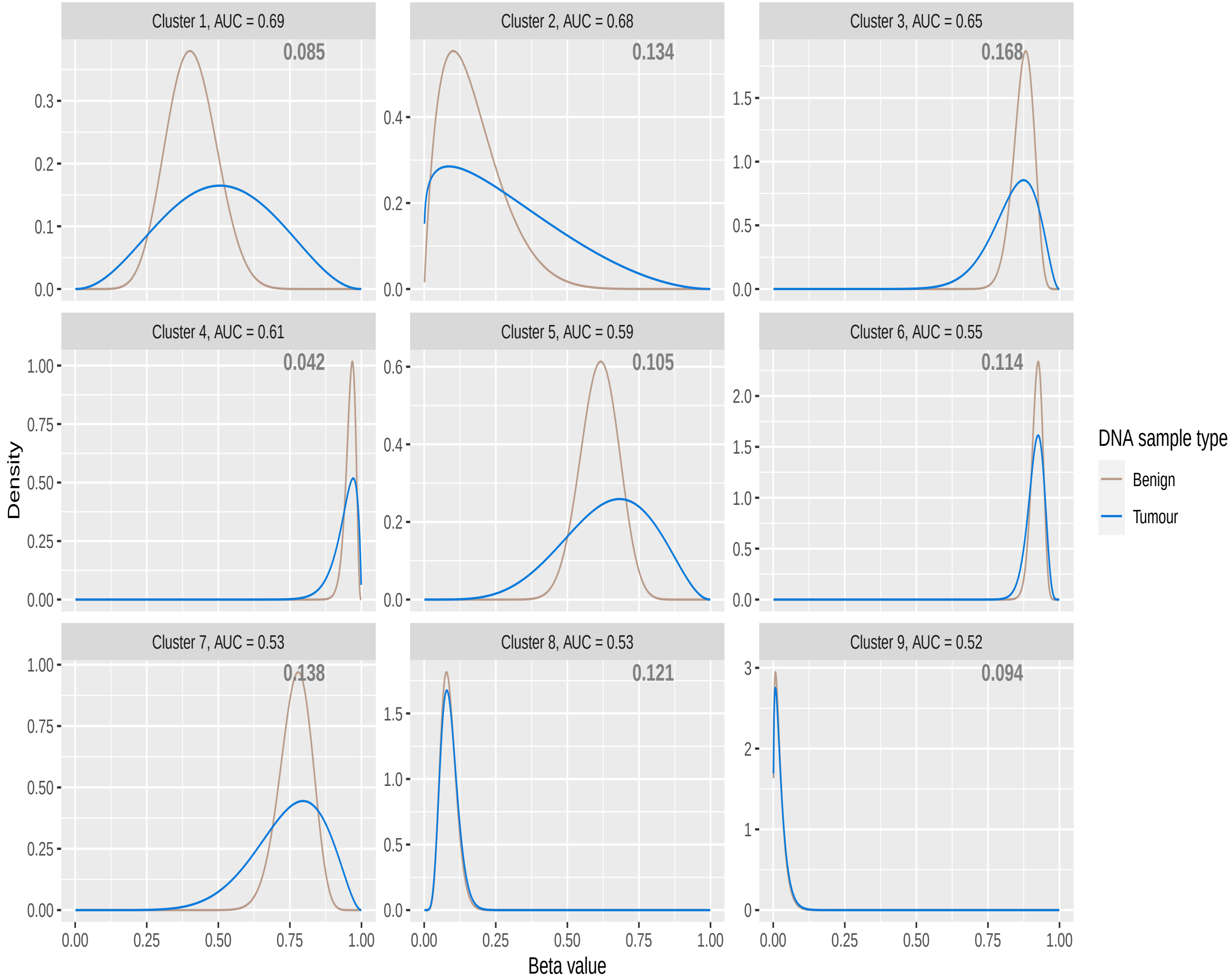

Figure 2: Fitted density estimates under the clustering solution of the K·R model for DNA methylation data from benign and tumour ESCC samples. The estimated mixing proportions are displayed in the relevant panel.

References

[1] Chen Y, Liao L, Wu Z, Yang Q, Guo J, He J, et al. Identification of key genes by integrating DNA methylation and next-generation transcriptome sequencing for esophageal squamous cell carcinoma. Aging (Albany NY). 2020;12(2):1332.

[2] Maksimovic J, Oshlack A, Phipson B. Gene set enrichment analysis for genome-wide DNA methylation data. Genome Biology. 2021;22(1):1-26.

[3] Lin Y, Zhang Y, Chen Y, Liu Z. Promoter methylation and clinical significance

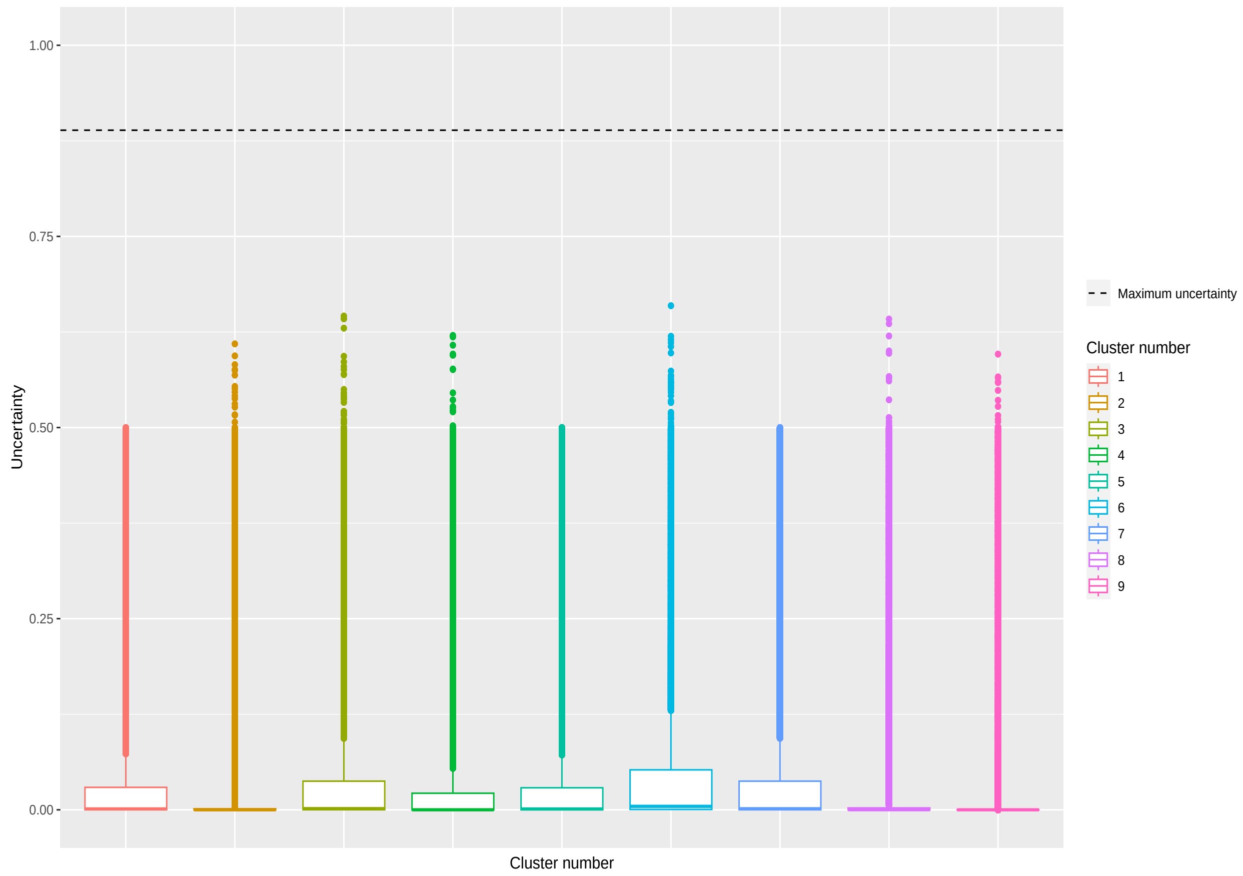

Figure 3: Clustering uncertainties for CpG sites in each clustering group under the clustering solution of the K·R model for the ESCC dataset.

of GPX3 in esophageal squamous cell carcinoma. Pathology-Research and Practice. 2019;215(11):152676.

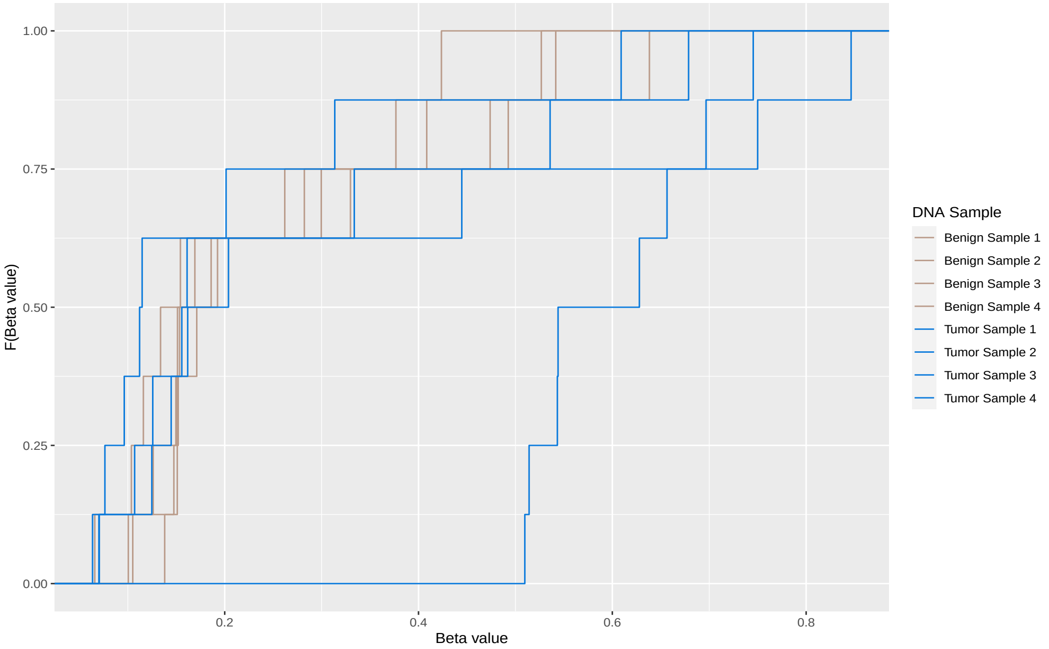

Figure 4: ECDFs for all the CpG sites identified as mostly differentially methylated and related to the GPX3 genes for all patient samples.
